# Supplementary material for: Two Types of Liposomal Formulations Improve the Therapeutic Ratio of Prednisolone Phosphate in a Zebrafish Model for Inflammation
Source: Cells. 2022 Feb 15;11(4):671. doi: 10.3390/cells11040671 (PMC8870436; doi:10.3390/cells11040671)
Supplement: Supplementary file 1 [file cells-11-00671-s001.zip › cells-1564472-supplementary.pdf]

Supplementary data

Supplementary Table S1. Physicochemical characterization of liposomes. Hydrodynamic radius and polydispersity index as determined by Dynamic Light Scattering, values of surface charge as determined by Zeta Potential measurements, and EE% calculated based on theoretical values and UV-VIS measurements (see Materials and Methods).

Liposomes	Size (nm)	PDI	Zeta potential (mV)	EE% Theoretical*	EE% measured
AmbiMACs (10% DSPG)	± 88.2	0.111	-36.9		
AmbiMACs (20% DSPG)	± 113.9	0.065	-41.0		
AmbiMACs (20% DSPG) *containing PLP*	± 117.3	0.052	-35.2	4.9	1.9
AmbiMACs (30% DSPG)	± 113.0	0.049	-41.8		
PEG liposome	± 114.0	0.051	-11.0		
PEG liposome *containing PLP*	± 131.1	0.269	-12.2	4.9	0.7

*****Liposome total internal volume (*Φinner*) roughly calculated for 1mL and 1mM liposomes: 10^{-6}mol x 6.23 x 10^{23} (N_A) = 6.023 x10^{17} lipids. Given that ~80.000 lipids comprise 1 liposome of about 100nm in size, then 6.023 x10^{17} lipids comprise 7.528 x 10^{12} liposomes. The (inner) volume of the liposome is the volume of a sphere with about 50nm radius : 4π/3 x r^3 = 4π/3 x $(50\times10^{-9}m)^3$ = 5.23 x 10^{-16} cm^3/liposome. Therefore, 5.23 x 10^{-16} x 7.528 x 10^{12} = **3.94 x 10^{-3} cm^3 total inner volume**. Calculations do not take into account membrane thickness and size dispersity.

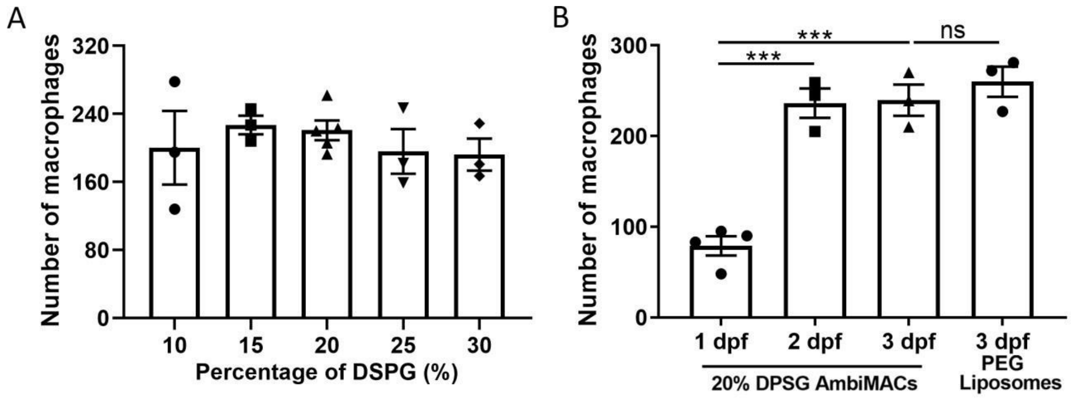

Supplementary Figure S1. The total number of macrophages in the body of embryos. *Tg(mpeg1:GFP)* embryos were injected with liposomes containing different percentages of DSPG at 2 days post fertilization (dpf) (A) or AmbiMACs (20% DSPG) at 1, 2 and 3 dpf and PEGylated liposomes at 3dpf (B). Confocal microscopy images were taken at 2 hours post injection (hpi) and the number of macrophages over the whole body was quantified. Statistical analysis was performed by one-way ANOVA with Bonferroni's post hoc test. No significant differences were observed in panel A. In panel B, no significant difference was observed when injected with AmbiMACs (20% DSPG) and PEG liposome at 3 dpf. Upon injection with AmbiMACs (20% DSPG), at 2 dpf and 3 dpf significantly higher numbers of macrophages were observed than at 1 dpf. Data shown are the means ± s.e.m. of 3-5 individual embryos, of which the individual data are indicated. Statistically significant differences between groups are indicated by: ns, non-significant; * $p<0.05$; **** $p<0.0001$.

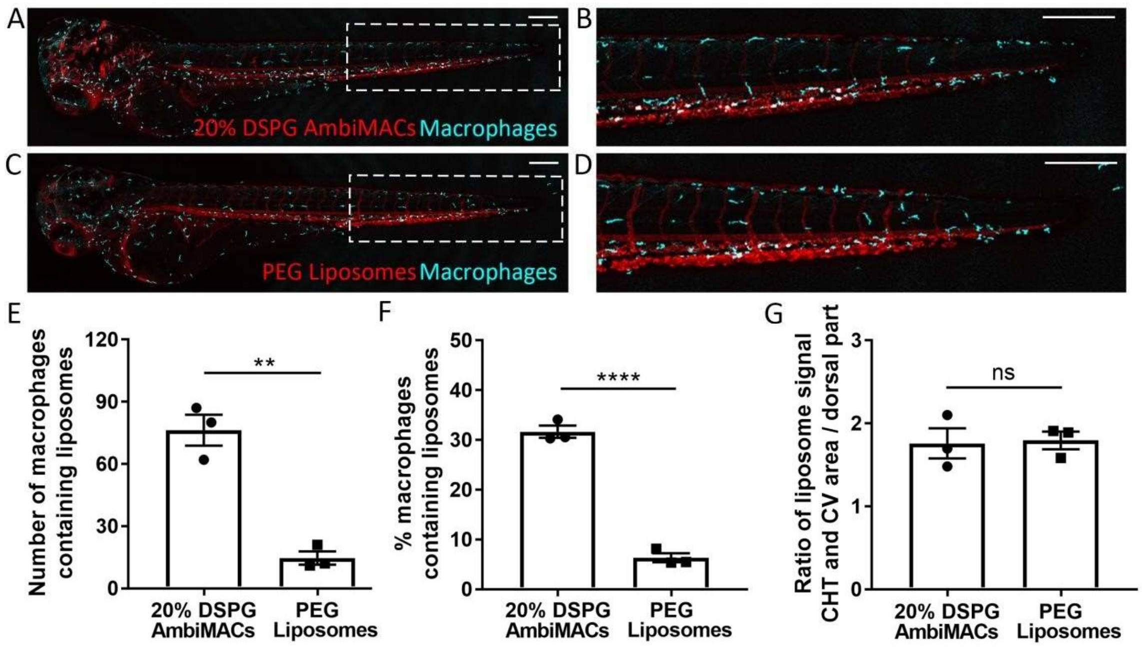

Supplementary Figure S2. Biodistribution in zebrafish embryos of liposomes encapsulating PLP. A-D. Representative confocal microscopy images of 3 dpf embryos of the *Tg(mpeg1:GFP)*line injected with AmbiMACs (20% DSPG) (A, B) or PEGylated (C, D) liposomes encapsulating PLP. Images were taken at 2 hpi. Liposomes are shown in red and macrophages in cyan. The tail regions (indicated by dashed boxes in A and C) are shown at higher magnification in B and D. Scale bar = 200 µm. E-G. The number (E) and percentage (G) of macrophages containing liposomes quantified in the whole body. Significantly lower number and percentage of macrophages containing liposomes were observed in the PLP encapsulated AmbiMACs (20% DSPG) injected embryos compared to the PLP encapsulated PEG liposomes injected group. I. The ratio between the (fluorescent) signal of liposomes in the CHT and CV area and the dorsal part (as described in Figure 1I). No significant difference was observed. Statistical analysis was performed by two-tailed t-test. Data shown are the means ± s.e.m. of 3 individual embryos, of which the individual data are indicated. Statistically significant differences between groups are indicated by: ** $p<0.01$; **** $p<0.0001$.

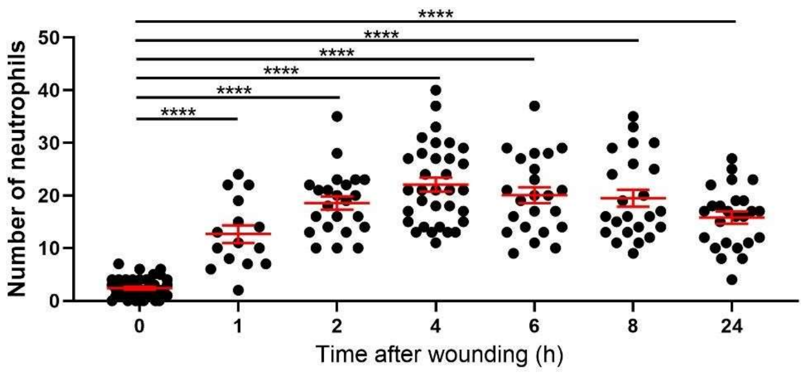

Supplementary Figure S3. Number of neutrophils recruited to the wounded area at different time points after laser wounding. Embryos (at 3 dpf) of the *Tg*(*mpx:GFP*) line were subjected to laser wounding, and fluorescence microscopy images were taken at different time points after the wounding procedure. The number of neutrophils recruited to the wounded area are shown. Statistical analysis was performed by one-way ANOVA with Bonferroni's post hoc test. Each data point represents a single embryo and the means ± s.e.m. of data accumulated from three independent experiments are shown in red. Statistically significant differences between groups are indicated by: **** p<0.0001.

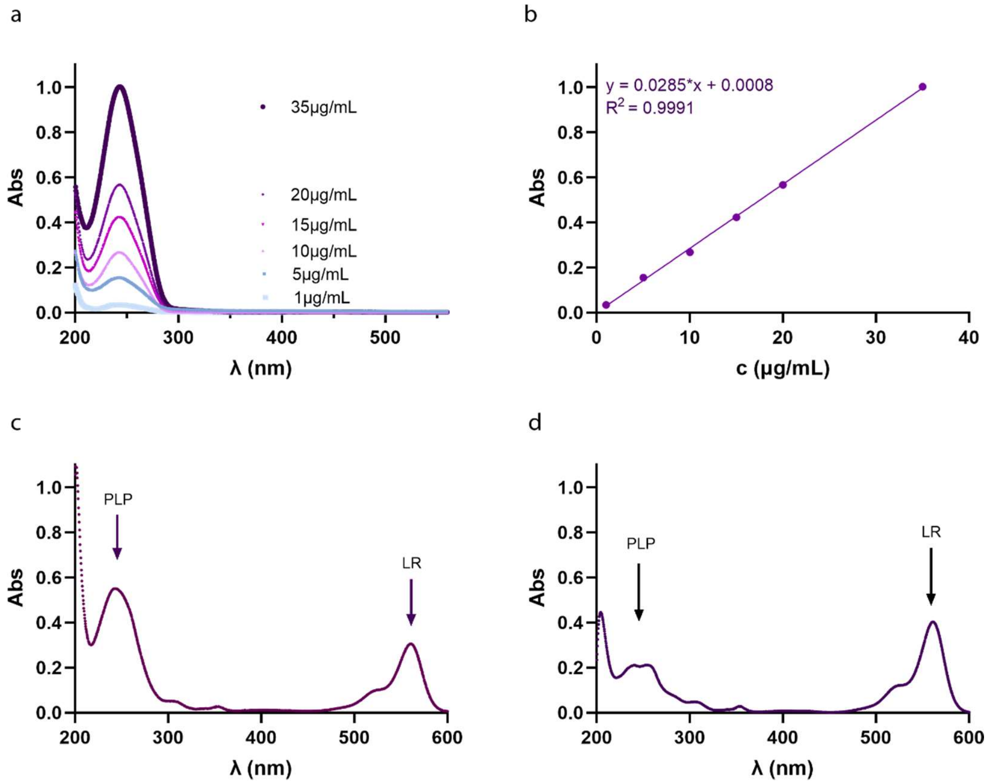

Supplementary Figure S4. UV-Vis measurements of prednisolone phosphate. (a) Absorbance of prednisolone phosphate at 242 nm at various concentrations (solution in MeOH) and (b) the corresponding calibration curve. (c) Detection of PLP in AmbiMAC liposomes after membrane disruption by dilution in methanol (20x). The peak at 565 nm corresponds to Lissamine Rhodamine PE incorporated in the liposome membrane (1% mol). (d) Detection of PLP in PEG liposomes after membrane disruption by dilution in methanol (20x). Peak at 565 nm corresponds to Lissamine Rhodamine PE incorporated in the liposome membrane (1% mol).

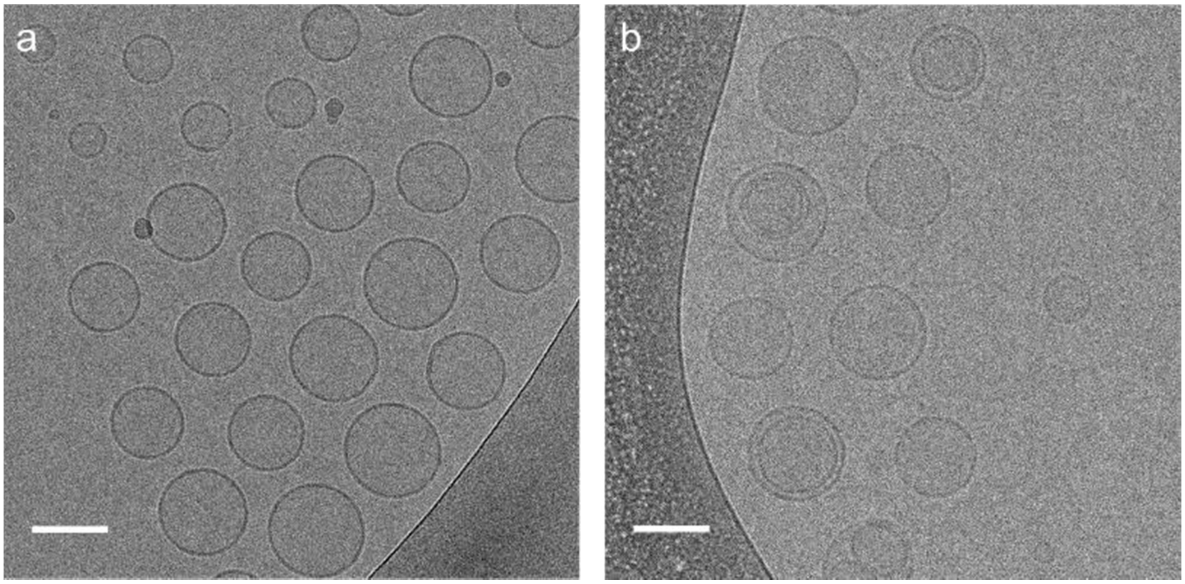

Supplementary Figure S5. CryoTEM of AmbiMAcs 20% DSPG. Liposomes as observed in cryoEM before (a) and after (b) prednisolone phosphate (PLP) encapsulation.
